# Comparison of endogenous hormone content and balance in *Pinus yunnanensis* Franch. seedlings after decapitation

**DOI:** 10.3389/fpls.2025.1531575

**Published:** 2025-04-16

**Authors:** Zhaoliu Hu, Sili Cheng, Bin He, Guangpeng Tang, Lin Chen, Shi Chen, Junrong Tang, Yulan Xu, Genqian Li, Nianhui Cai

**Affiliations:** ^1^ Resources Conservation and Utilization in the Southwest Mountains of China, Ministry of Education, Kunming, China; ^2^ National Forestry and Grassland Administration on Biodiversity Conservation in Southwest China, Southwest Forestry University, Kunming, China; ^3^ Communications Vocational and Technical College, Party Committee Inspection Office, Kunming, Yunnan, China

**Keywords:** decapitation, *Pinus yunnanensis* Franch., plant regulators, tillering development, hormonal balance

## Abstract

*Pinus yunnanensis* is a pioneer tree species and an important timber tree species for afforestation in barren hills in southwest China. It can improve the degradation of *P. yunnanensis* germplasm resources by decapitation to get high-quality spikes to establish a cutting nursery. The growth and development of sprouting tillers is the result of coordinated regulation of various endogenous hormones, and finally develops into spikes. We aimed to investigate the content changes of endogenous hormones indole-3-acetic acid (IAA), gibberellin_3_ (GA_3_), zeatin (ZT), and abscisic acid (ABA) in *P. yunnanensis* seedlings after decapitation, and to lay a foundation for hormone regulation mechanism in combination with sprouting ability. The plant were one-year-old *P. yunnanensis* seedlings, the hormone variation characteristics were clarified by decapitation to promote sprouting, and combined with the sprouting ability to analyze the endogenous hormone variations. Decapitation significantly improved GA_3_, ZT+GA_3_, IAA+ZT+GA_3_, and the early sprouting stage (ESS) of IAA and ABA. ZT was no significant change. Decapitation significantly improved the GA_3_/ABA, (ZT+GA_3_)/ABA, (IAA+ZT+GA_3_)/ABA and the ESS of IAA/ABA, and significantly reduced ZT/IAA in the ESS responded decapitation by changing the contents and the balance. The changes in dynamic balance in GA_3_, GA_3_/ABA, (ZT+GA_3_)/ABA, and (IAA+ZT+GA_3_)/ABA were the most significant. The sprouts number was significantly positively correlated with GA_3_, ZT+GA_3_, IAA+ZT+GA_3_, GA_3_/ABA, (ZT+GA_3_)/ABA, (IAA+ZT+GA_3_)/ABA, and significant positive correlation with ZT/IAA. Sprouts growth was extremely significantly positively correlated with GA_3_, GA_3_/ABA, (ZT+GA_3_)/ABA, and (IAA+ZT+GA_3_)/ABA. The hormone contents and ratios regulated the occurrence and germination of growth. Dynamic balance of GA_3_, GA_3_/ABA, (ZT+GA_3_)/ABA, and (IAA+ZT+GA_3_)/ABA played more important roles in the number and growth of sprouts. GA_3_ played a crucial regulatory role in promoting the sprouting and growth. IAA, ZT, and ABA played an important regulatory role through the interaction between hormones. The mutual balance of hormones promotes the growth and development of sprouting tillers of *P.yunnanensis*. This experiment explored the response of endogenous hormone content and ratio changes to tiller sprouting by comparing decapitation and non-decapitation, explored the growth and development law of *P. yunnanensis* tiller sprouting, shortened the seedlings cycle, and provided a scientific basis for the establishment of cutting nursery.

## 1 Introduction

The germination of axillary buds can be divided into four stages: axillary bud initiation and formation, associated inhibition (apical dominance), induction (bud activation), and sustained growth leading to axillary branching (Tan et al., 2019). Each of these stages is affected by various hormones (Waldie et al., 2010). Emergent tiller genesis and lateral branch growth are closely related to phytohormones and signal transduction processes (Barbier et al., 2019). Phytohormones can initiate downstream signaling and regulate gene expression to control lateral branch emergence or dormancy (Dong et al., 2023), and perform a pivotal function in regulating plant meristem development (Shen et al., 2019). The hypothesis of plant germination regulation holds that plants’ germination and regeneration ability is regulated by the internal level (Wanze et al., 2007). It can be seen that the ability of plants to sprout is closely related to hormone levels. Indole-3-acid (IAA), gibberellin (GAs), and cytokinin (CTK) are considered to play a significant role in the regulation of plant sprouting characteristics (Katyayini et al., 2020; Müller and Leyser, 2011). Later studies have shown that Strigolactone (SL), Brassinolide (BRs), Abscisic acid (ABA), and sugar can regulate plant branching development (Yuan et al., 2023), but the regulatory mechanism remains unclear. The study results proved that the development and germination after decapitation were relevant to hormonal regulation (Liu et al., 2011). Decapitation regulates sprout regeneration by regulating the balance of endogenous hormones in dormant buds of felling stumps (Eliassion, 1971). There are two classical hypotheses about auxin regulating plant lateral development, the auxin transport channel hypothesis and the second messenger hypothesis. After decapitated plants, the auxin from the top disappears, the auxin transport capacity in the main stem is improved, export of auxin from the dormant bud to the stem is initiated, and the dormant bud is activated (Domagalska and Leyser, 2011), auxin controls plant branching development by regulating hormone levels of cytokinins and strigolactones (Bangerth et al., 2000). It can be seen that the regulation of auxin on axillary bud growth is indirect. The cytokinins transported from roots to stem increased after the aboveground plant parts were damaged, high concentration of cytokinins promoted germination of stems and the formation of branches (Schmülling, 2002). The high cytokine content in *Cicer arietinum* germination is closely related to its strong sprouting ability (Turnbull et al., 1997). The increase of GA_3_ content after the decapitation of *Jatropha curcas* exerted a crucial part in promoting the breaking of dormant buds, which was related to the germination and growth of lateral buds (Ni et al., 2015). Overexpression of GA20 oxidase (*GA2ox*), a GA catabolic gene, led to an increase in the number of tillers or branches. GA level was negatively correlated with the formation of axillary meristem or tillers (Martínez-Bello et al., 2015). Therefore, the regulation of gibberellin branch growth of different species may be different. The increase of ABA inhibited the development of lateral buds, and ABA content negatively regulates lateral branching at later stages (González-Grandío et al., 2017). Therefore, ABA is considered to negatively regulate branch growth. Changes in hormone content and distribution after decapitation have differences in the growth and development of sprouting, thus affecting the germination ability. The regulation of dormant buds should not only consider the influence of content but also consider the influence of balance, especially the ratio and balance between promoting and inhibiting bud germination (Beveridge et al., 2003; Shaolin, 2020).

The interaction of various hormones in plants can produce an extremely complex regulatory system, which regulates many metabolisms of plant growth and development (Leitão and Enguita, 2016). Some receptors or key components in the hormone signal transduction system will produce synergistic or antagonistic effects due to interaction or crosstalk, which makes the signal pathway networked (Ohri et al., 2015). IAA can regulate the synthesis of GAs and their signal transduction. Cytokinins are involved in the signal transduction of strigolactones and regulate the level of cytokinins by inhibiting the expression of isopentenyl transferases, a key gene of cytokinin synthase. GA signal regulates strigolactone biosynthesis by regulating gene expression (Ito et al., 1999; Lv et al., 2018). Auxin, cytokinin, and gibberellic acid independently and in combination to regulate the function of meristem (Depuydt and Hardtke, 2011). Therefore, plant hormones regulate plant growth and development by forming an interaction network between upstream and downstream signals. Except for the plant hormones, plant branches are also regulated by sucrose, light, and nutrition (Rameau et al., 2014). Plant growth and development are regulated by both internal factors and external environment. Internal factors include genetic factors, sugars, and hormones. Environmental factors include nutrients, water, and photoperiod (Kebrom et al., 2013). When the external environmental factors change, plant hormones trigger the downstream signal transduction and regulate the expression level of genes, thereby regulating the dormancy or germination of buds and environmental regulation of tiller bud elongation (Dong et al., 2023). The crosstalk between plant hormones, carbohydrates, and other signal transductions synergistically promoted the germination of *Pinus echinata* after decapitation (Liu et al., 2011). Therefore, the regulatory network of branching involves a variety of types of participants (plant development, genotype, hormones, nutrition). Its interactions with the feedback regulation of buds and plants, the fluctuations of system dynamics, and environmental variables related to plant development make this process more complicated (Rameau et al., 2014). It is very challenging to understand the regulation of branching (Barbier et al., 2019).

*Pinus yunnanensis* Franch. is an evergreen arbor of the genus Pinus and subgenera Pinus in the Pinaceae family. It is an important timber tree species in southwest China and a pioneer tree species for mountain afforestation. Under the condition of natural regeneration, the seedling growth of *P. yunnanensis* was slow, and the stem shape declined. Genetic improvement and decapitation rejuvenation are important ways to change this situation. After decapitation, it can promote the germination of lateral buds or hidden buds, and can obtain young spikes for asexual reproduction, and the reproduction coefficient is significantly improved. Therefore, the regulation characteristics of sprouting hormones in *P. yunnanensis* seedlings after decapitation are an important basis for the study of seedlings sprouting promotion. At present, the study on endogenous hormone content after decapitation of *P. yunnanensis* seedlings is relatively rare. How does endogenous content change after decapitation affect the growth and development of sprouting tillers? This study obtained one-year-old *P. yunnanensis* seedlings as research materials by cultivating seedlings in the greenhouse. The decapitation treatment was set (the height from the root neck to the top of 10cm), and the non-decapitation plant as control. The response of the law about sprouting and growth of *P. yunnanensis* to stump height was studied based, on the relationship between hormone changes and sprouting ability and was taken as the core to clarify the change rule of endogenous hormone content level after decapitation. The analysis of the response of endogenous hormone content to decapitation and the causal relationship between endogenous hormone characteristics and sprouting ability, lays a foundation for exploring the hormone regulation mechanism of sprouting ability. To reveal the physiological mechanism of hormone promoting the growth and development of sprout tillers of *P. yunnanensis* after decapitation, and to obtain high-quality cuttings to establish a high-quality cutting nursery. The asexual reproduction method of cuttings can effectively retain the excellent traits of the mother plant, shorten the breeding cycle, improve the breeding efficiency, and improve the quality of *P. yunnanensis*.

## 2 Materials and methods

### 2.1 Study area

The experimental site is located in Maitreya City, Honghe Hani and Yi Autonomous Prefecture, Yunnan Province, and is located in the north of Honghe Prefecture in the southeast of Yunnan Province (101°47′-104°16′ E, 22° 26′-24° 45′ N). This area belongs to a subtropical monsoon climate, adequate illumination, a long effective temperature period, and short frost and snow days. The average annual temperature is 17.1°C, The average annual precipitation is 950.2 mm, the relative humidity is 73%, the number of sunshine hours is 2131.4 hours, and the frost-free period is 323 days.

### 2.2 Plant material

The seeds of *P. yunnanensis* were collected from the Midu Clonal Seed Orchard of *P. yunnanensis* (Yun S-CSO-PY-001-2016). In March 2018, the seedlings were cultivated in the nursery base of Jicheng Landscape Technology Shares Limited in Maitreya City, Honghe Prefecture, Yunnan Province. A single plant was transplanted into the seedlings pot (Base diameter 16 cm/caliber 24 cm/height 20 cm) after 3 months. Seedling management was mainly watering and weeding, watering once every 3-5 days to water thoroughly. Seedling management is chiefly watering and weeding, watering once in 3-5 days to water thoroughly, weeding once a month, remove all weeds in a seedling pot. The field experiment was designed by a single factor completely randomized block experiment design. According to the results of the pre-experiment, two treatments were applied: (1) Decapitation treatment: Cut the top with scissors, and maintain a stem height of 10 cm (abbreviated as +DEC). Paint on the top after decapitation to avoid water evaporation and microbial interference. (2) Treatment of non-decapitation as control (abbreviated as -DEC). Each treatment was repeated 3 times, 2 plots per replicate, a total of 6 plots, 40 seedlings per plot, and a total of 240 seedlings. The management of seedlings after truncation is the same as before. The average seedling height before decapitation is 20 cm and the average ground diameter is 17 mm. Before decapitation, *P. yunnanensis* seedlings with strong growth and relatively consistent height were selected and stumped at the end of March 2019.

### 2.3 Dynamic observation of sprouting branches

Background investigation before test layout and follow-up investigation after test layout. The investigation included the number of sprouting branches, the growth of sprouting branches, and the growth of non-decapition plants. The survey was conducted at the end of each month from March 2019 to December 2019. Investigate each sprout and count the number of sprouting branches. There were three replicates in each sample group, and 10 seedlings were randomly selected from each replicate to track and investigate the growth of sprouting tillers. The growth of 30 seedlings in each treatment group was statistically investigated. Due to the large number of sprouting branches, the majority of them were very short and rarely were the potentially effective sprouting branches with lengths larger than 1.5 cm investigated. The height of control seedlings was measured at the same time.

### 2.4 Sample collection and hormone content determination

In April, May, June, and late July 2019, mixed sampling was performed on the sprout needles of the decapitated and control groups of *P. yunnanensis*, and each sampling from the fixed 5 plants was a biological repeat, with a total of 3 replicates. Two treatment groups were sampled respectively. Latex gloves and masks were worn during collection to minimize sample contamination. Immediately after sampling, the mixed needle samples were placed in a 5 ml centrifuge tube and labeled, then quickly placed in liquid nitrogen and brought back to the laboratory for storage in an ultra-low temperature refrigerator at -80 °C for endogenous determination. The post-processing method of sample collection is based on the operation method of Li et al (Li et al., 2016). The liquid phase conditions after the sample were performed as described by Hui et al (Hui et al., 2018).

### 2.5 Data analysis

The contents and ratios of endogenous IAA, ZT, GA_3_, and ABA were sorted out by Excel software, one-way analysis of variance was performed using SPSS27.0, and Duncan *Post Hoc* test was used for significance test (*P* < 0.01, *P* < 0.05). The number of sprouting branches, the growth of sprouting branches, hormone content and ratio were correlated. The effects of hormone content and its ratio on the number and growth of sprouts were judged according to the significant level. The relationship between sprouting ability and the hormones of *P.yunnanensis* was analyzed by correlation analysis and path analysis. The regression analysis of hormones and seedling height growth was carried out to reveal the effect of hormones on high growth. The software used was SPSS27.0, Excel2019, GraphPad Prism 10, Origin2021 Pro.

## 3 Results

### 3.1 Decapitation can promote the sprouting ability of *P. yunnanensis*

**Figure 1:**
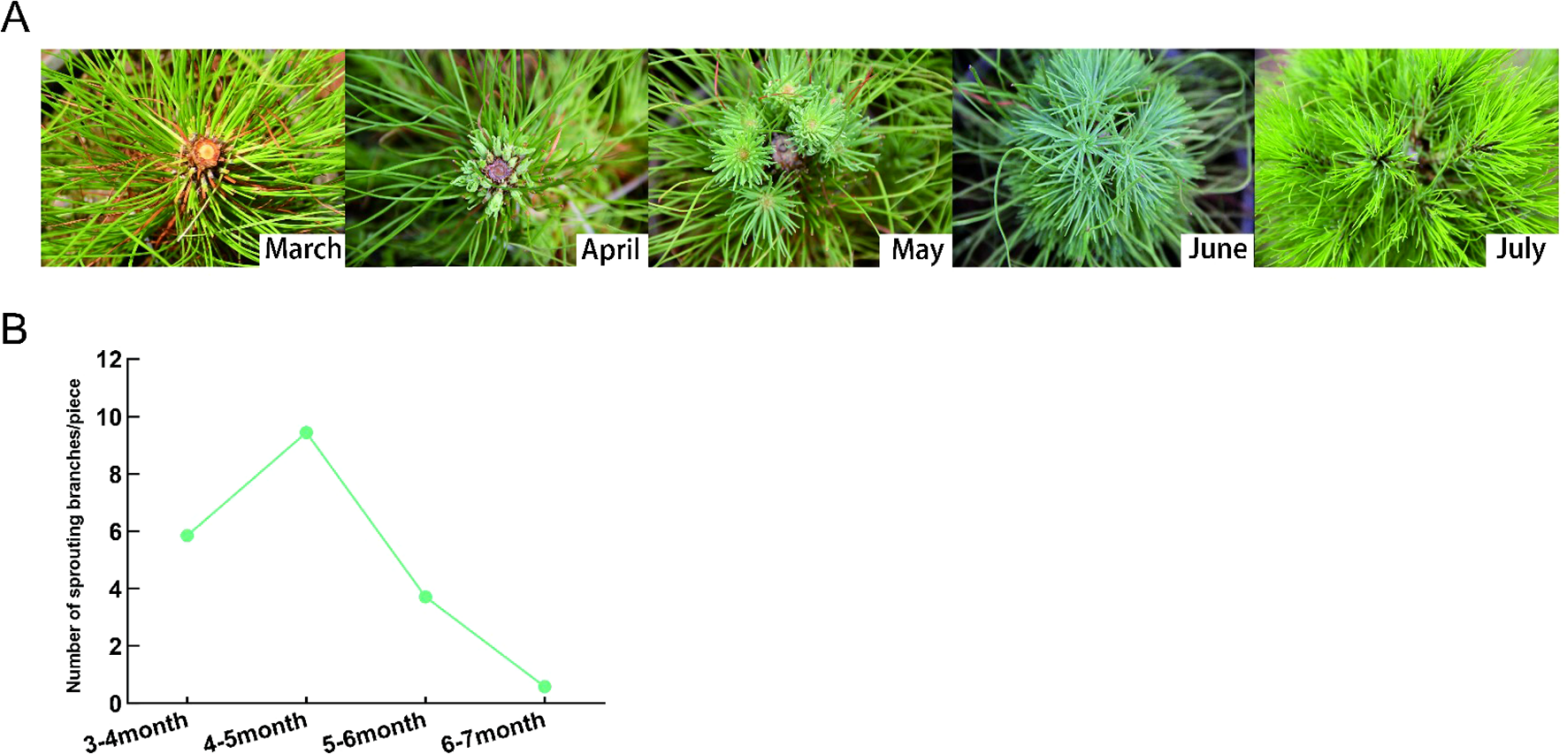
Sprouting ability of Yunnan pine after decapitation. **(A)** The sprouting branch of *P. yunnanensis* changes with time after decapitation. **(B)** The number of sprouting branch at different times after decapitation.

The sprouting ability of *P. yunnanensis* seedlings is weak under natural conditions, while decapitation can effectively promote the sprouting of *P. yunnanensis* seedlings. It can be seen that from April to July after decapitation, the axillary buds of *P. yunnanensis* germinated rapidly and formed lateral branches (**Figure 1A**). After decapitation, the axillary buds of *P.yunnanensis* germinated rapidly to form lateral branches in April, and the trend increased rapidly at first and then decreased gradually (**Figure 1B**).

### 3.2 Endogenous hormone content after decapitation

**Figure 2:**
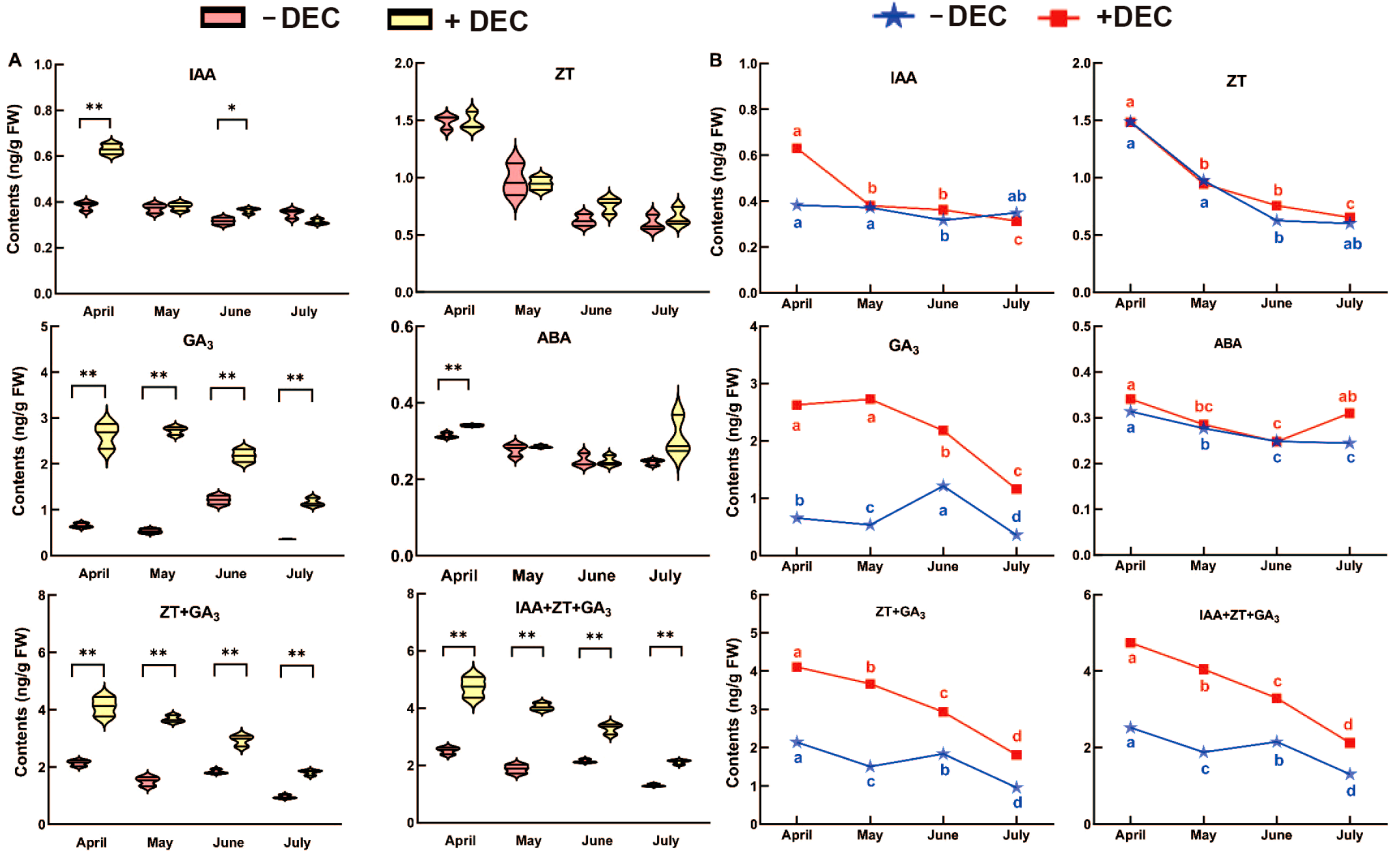
The change rule with time of the endogenous hormone content for *P. yunnsnensis* seedlings after decapitation and non-decapitation. **(A)** The significant difference between non-decapitation and decapitation treatments. The error bars represent standard error. ‘*’ on error bars indicate significant differences at *p* < 0.05, ‘**’ on error bars indicate significant differences at *p* < 0.01. **(B)** The different small letters in the same treatment indicate the significant difference among the different months at *p* < 0.05. Decapitation: +DEC; Non-decapitation: -DEC.

As shown in **Figure 2A**, at the same time, decapitation was significantly the of GA_3_, ZT+GA_3_, IAA+ZT+GA_3_ in the whole sprouting branch, while ABA was only significantly higher in April. After decapitation, the IAA was significantly higher than the non-decapitation in April and June. There was no significant difference in ZT after decapitation. As shown in **Figure 2B**, GA_3_ and IAA decreased with time after decapitation. GA_3_ increased significantly in June and IAA increased in July in non-decapitation plants. ABA decreased with time in different treatments, and ABA increased significantly in July after topping. After decapitation, ZT, ZT+GA_3_, IAA+ZT+GA_3_ gradually decreased with time. In non-decapitation plants ZT+GA_3_ and IAA+ZT+GA_3_ increased in June compared with May. The results showed that decapitation significantly alters the hormone levels of GA_3_, ZT+GA_3_ and IAA+ZT+GA_3_ content, in which these hormones can be involved in the process of sprouting and growth after decapitation.

### 3.3 The balance of endogenous hormones after decapitation

**Figure 3:**
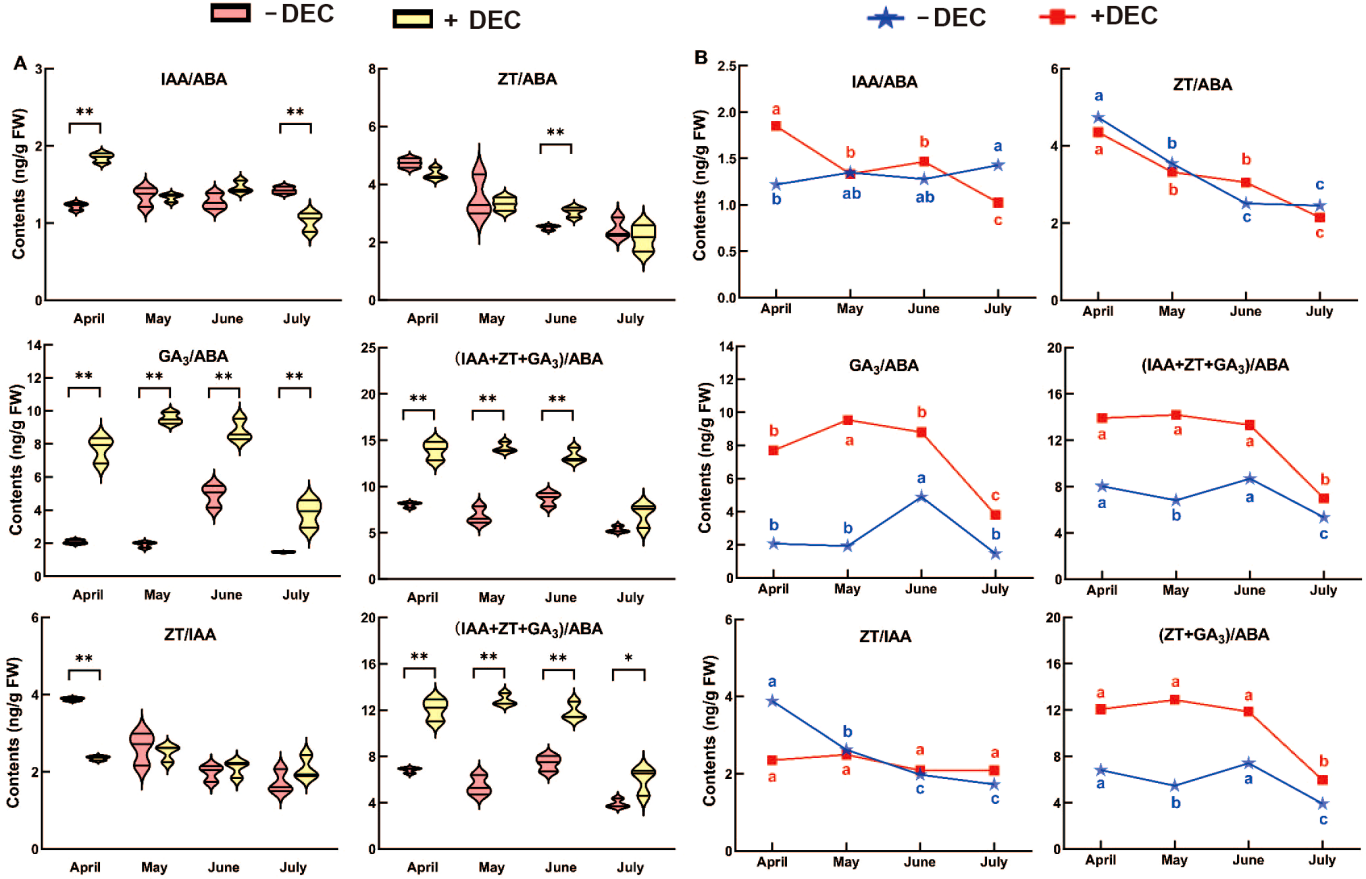
The change rule with time of the endogenous hormones ratio for *P. yunnsnensis* seedlings after decapitation and non-decapitation. **(A)** The significant difference between non-decapitation and decapitation treatments. The error bars represent standard error. ‘*’ on error bars indicate significant differences at *p* < 0.05, ‘**’ on error bars indicate significant differences at *p* < 0.01. **(B)** The different small letters in the same treatment indicate the significant difference among the different months at *p* < 0.05.

After decapitation compared with the non-decapitation at the same stage (**Figure 3A**), the balance of GA_3_/ABA, (IAA+ZT+GA_3_)/ABA, (ZT+GA_3_)/ABA were significantly increased during the sprouting period. The IAA/ABA in decapitation treatment group was significantly higher in April and lower in July than that non-decapitation. After decapitation, ZT/ABA in June was significantly higher than that non-decapitation, and decapitation was lower in April, May and June. After decapitation, ZT/IAA was significantly higher than that non-decapitation in April. As shown in **Figure 3B**, ZT/IAA and ZT/ABA decreased with time in different treatments. After decapitation, (IAA+ZT+GA_3_)/ABA and (ZT+GA_3_)/ABA decreased significantly in July, increased significantly in June, and then decreased in non-decapitation plants. After decapitation, (IAA+ZT+GA_3_)/ABA and (ZT+GA_3_)/ABA decreased significantly in July, and increased significantly in June and then decreased in non-decapitation plants. After decapitation, IAA/ABA decreased with time, GA_3_/ABA increased first and then decreased in May, IAA/ABA increased with time in non-decapitation plants, and GA_3_/ABA increased significantly in June.

In summary, the balance of GA_3_/ABA, (ZT+GA_3_)/ABA, and (IAA+ZT+GA_3_)/ABA was significantly increased during the whole sprouting process, the IAA/ABA was significantly increased at the early stage, and the ZT/IAA was significantly decreased at the early stage. Decapitation changed the time trend of hormone balance, except for the ZT/ABA. The results showed that the dynamic balance of these hormones may be involved in sprouting and growth after decapitation.

### 3.4 Response of sprouting branches number to hormone content and balance

**Figure 4:**
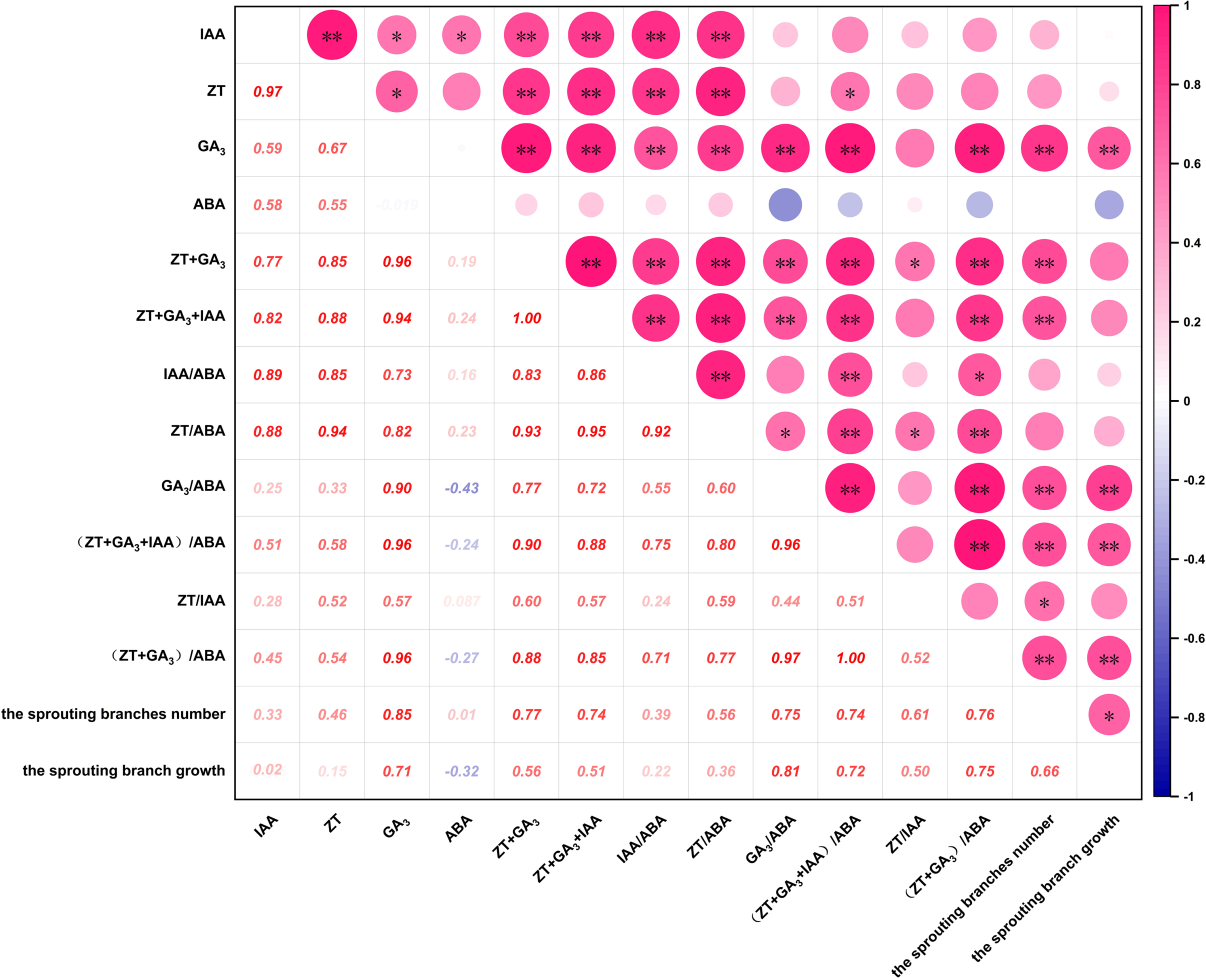
Correlation analysis of hormones with the sprouting branches number and the sprouting branch growth of *P. yunnsnensis.* ‘*’, *p* < 0.05; ‘**’, *p* < 0.01.

As shown in **Figure 4**, The number of sprouting branches was positively correlated with the contents of ZT, IAA and ABA, and was significantly positively correlated with the contents of GA_3_, ZT+GA_3_ and IAA+ZT+GA_3_. The results that the hormone content could promote the sprouting of *P. yunnanensis*, among which GA_3_, ZT+GA_3_ and IAA+ZT+GA_3_ could significantly promote the sprouting. There was a positive correlation between ABA and the number of sprouting branches, but the correlation coefficient was low, it could be ignored. It can be seen that the number of sprouting branches was positively correlated with IAA/ABA and ZT/ABA, significantly positively correlated with ZT/IAA, and extremely significantly positively correlated with GA_3_/ABA, (ZT+GA_3_)/ABA and (IAA+ZT+GA_3_)/ABA. The results indicated that the hormone balance promoted the sprouting branches of *P. yunnanensis*, and the balance of GA_3_/ABA, ZT/IAA, (ZT+GA_3_)/ABA, and (IAA+ZT+GA_3_)/ABA significantly promoted the sprouting branches. Additionally, the dynamic balance of GA_3_, GA_3_/ABA, (ZT+GA_3_)/ABA, and (IAA+ZT+GA_3_)/ABA played a crucial regulatory role in the process of decapitation sprouting of *P. yunnanensis*.

The growth of sprouting branches was positively correlated with ZT, ZT+GA_3_, and IAA+ZT+GA_3_, significantly positively correlated with the contents of GA_3_ (*P* < 0.01), and negatively correlated with the contents of ABA. The results indicated that ZT, IAA, GA_3_, ZT+GA_3_, and IAA+ZT+GA_3_ could promote the growth of sprouting branches of *P. yunnanensis*, in which GA_3_ promoted significantly and ABA inhibited the growth of sprouting branches. The results showed that there was a positive correlation between the growth of sprouting branches and the balance of ZT/ABA, IAA/ABA, and ZT/IAA, and a significant positive correlation between the growth of sprouting branches and GA3/ABA, (ZT+GA_3_)/ABA and (IAA+ZT+GA_3_)/ABA. The results indicated that the balance of ZT/ABA, IAA/ABA, GA_3_/ABA, ZT/IAA, (ZT+GA_3_)/ABA, and (IAA+ZT+GA_3_)/ABA could promote the growth of sprouting branches of *P. yunnanensis*. The balance of GA_3_/ABA, (ZT+GA_3_)/ABA, and (IAA+ZT+GA_3_)/ABA could significantly promote the growth of sprouting branches. It can also be seen that the dynamic balance of GA_3_ and GA_3_/ABA, (ZT+GA_3_)/ABA, and (IAA+ZT+GA_3_)/ABA significantly promoted the growth of sprouting branches of *P. yunnanensis* after decapitation.

### 3.5 Correspondence of sprouting ability of *P. yunnanensis* to hormones

**Figure 5:**
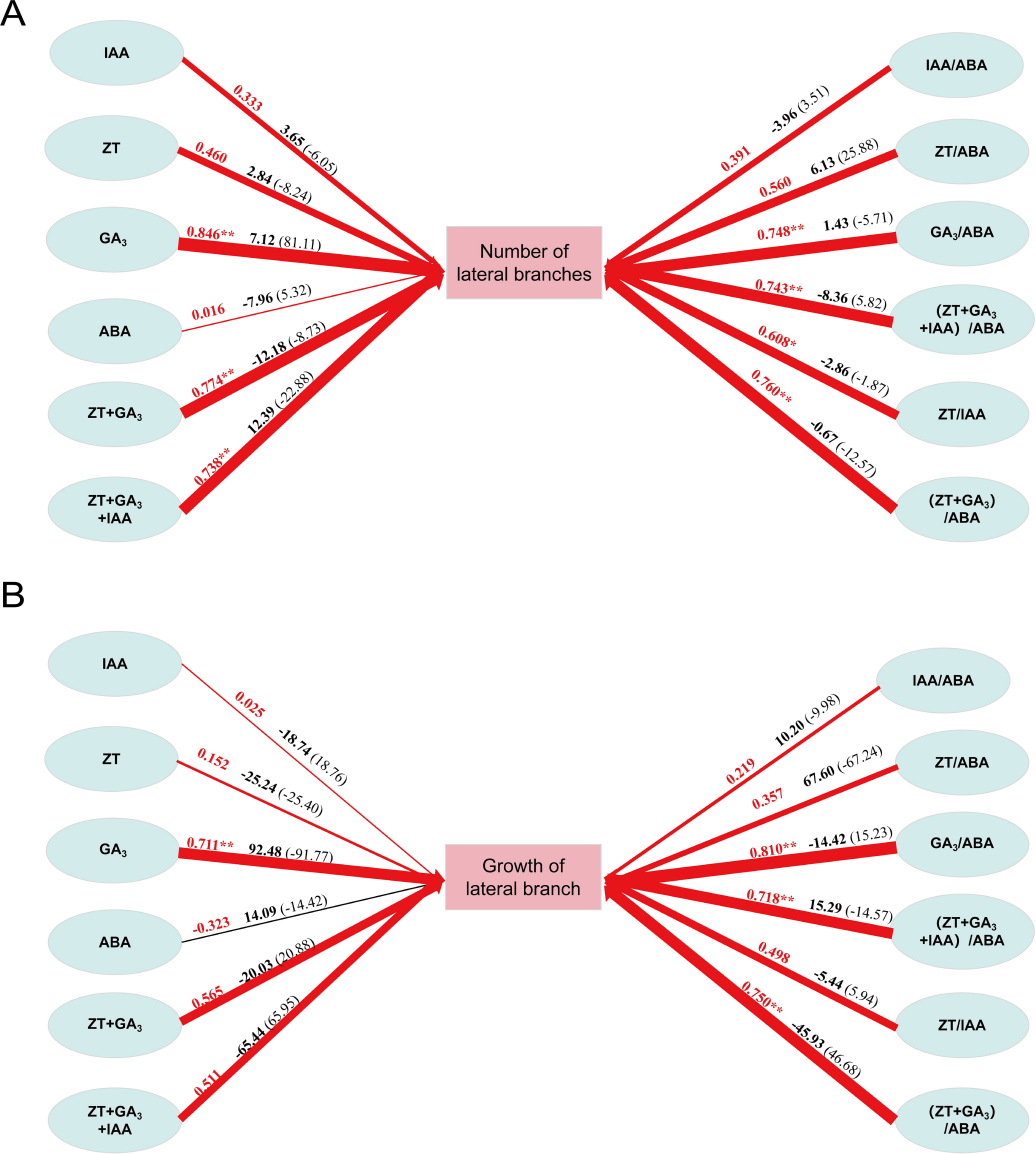
Path analysis of hormone and sprouting growth of *P. yunnanensis*. **(A)** Path analysis of hormones and the number of lateral branches; **(B)** Path analysis of hormones and lateral branch growth. The red arrow indicates that the path relationship is positively correlated, and the black arrow path relationship is negatively correlated. The arrow thickness represents the relative size of the normalized path coefficient. The red number on the arrow is the size of the correlation coefficient, the black bold character is the size of the direct path, and the number in the bracket is the size of the indirect path.

Through path analysis, it can be seen from **Figure 5A** that the direct path coefficient of IAA+ZT+GA_3_(12.39) was the largest, followed by GA_3_(7.12). The indirect effect of GA_3_(33.11) was the largest. The remaining hormone changes play an indirect role through GA_3_ and IAA+ZT+GA_3_. This shows that the changes of GA_3_ and IAA+ZT+GA_3_ content play a leading role in the change of the number of lateral branches of P. *yunnanensis*, and the changes of other hormone content play an indirect role in promoting the germination of lateral branches of P. *yunnanensi*s.

According to **Figure 5B**, the direct path coefficient of GA_3_(92.48) was the largest, followed by ZT/ABA (67.60). The indirect effect of IAA+ZT+GA_3_(65.95) was the largest. The remaining hormone changes play an indirect role through GA_3_ and ZT/ABA. The results showed that the changes of (ZT+GA_3_)/ABA and IAA+ZT+GA_3_ contents played a leading role in the change of the growth of lateral branches of *P.yunnanensi*s, and the changes of other hormone contents played an indirect role in promoting the growth and development of the lateral branches of *P.yunnanensis*.

### 3.6 Response of height growth of control seedlings to hormone content and balance

To understand the change rule of the relationship between seedling height growth (y) and hormone content (x) with time in non-decapitation, regression analysis was performed on the seedling height growth, hormone content, and hormone balance of the non-decapitation from April to July (**Table 1**). The height growth of control seedlings was positively correlated with hormone content (*P* > 0.05). The results showed that IAA, ZT, GA_3_, and ABA could promote the growth of seedling height, but the promotion effect was not significant. The results showed that IAA, ZT, GA_3_, and ABA could promote the growth of seedling height, but the effect was not significant. There was a negative correlation between seedling height growth and IAA/ABA (*P* > 0.05), and a positive correlation with the balance of other hormones (*P* > 0.05). The results showed that IAA/ABA inhibited the growth of seedling height, and other hormone balance promoted the growth of seedling height in non-decapitation, but the promotion and inhibition were not significant.

**Table 1:** Regression analysis of monthly growth of seedling heights and hormones.

Hormone content				Ratio of hormone			
Index	Regression equation	Correlation coefficient	Significance	Index	Regression equation	Correlation coefficient	Significance
(IAA+ZT+GA_3_)	y=0.419x+0.554	0.297	0.349	IAA/ABA	y=-2.070x+4.104	0.344	0.274
(ZT+GA_3_)	y=0.423x+0.697	0.297	0.351	(ZT+GA_3_)/ABA	y=0.114x+0.701	0.255	0.422
ABA	y=6.928x-0.330	0.292	0.358	(IAA+ZT+GA_3_)/ABA	y=0.111x+0.572	0.239	0.454
ZT	y=0.355x+1.055	0.200	0.532	ZT/IAA	y=0.171x+0.941	0.232	0.469
GA3	y=0.361x+1.128	0.182	0.570	ZT/ABA	y=0.113x+1.004	0.173	0.588
IAA	y=1.209x+0.947	0.055	0.862	GA_3_/ABA	y=0.065x+1.210	0.138	0.665

## 4 Discussion

Apical dominance can inhibit the growth of lateral buds, and decapitation is of great significance in promoting the germination and growth of plant lateral branches. Studies have shown that the apical dominance is relieved after decapitation, and the growth and development of sprouting are closely related to the changes of hormones (Cao et al., 2023; He et al., 2024; Shimizu-Sato et al., 2009). However, as the main tree species in rural areas of China, there are few studies on the physiological mechanism of hormones promoting the growth and development of sprouting after decapitation. In this study, the morphological indexes of seedlings decapitation and non-decapitation were counted, and the changes of hormone content in seedlings after combined treatment were clarified, and the physiological mechanism of hormones promoting the growth and development of sprouting was clarified.

This study showed that *P. yunnanensis* responded to hormone content and balance regulation after decapitation. There was a central system in plants that caused changes in hormones when stressed by adversity (Chapin, 1991). After decapitation, the aboveground tissue of the plant was destroyed, causing a change in hormone content in the body (Mitchell, 1992), which in turn affects the relative content of hormones. The growth and development of plants require coordinated control of multiple hormones (Santner et al., 2009). The type and time of operation are different, different hormones have different effects on the rooting of mulberry cuttings, and the time of hormone treatment is also the main factor affecting the rooting of mulberry (Sun et al., 2023). In our study decapitation significantly increased the contents of GA_3_, ZT+GA_3_, and IAA+ZT+GA_3_ in the process of sprouting and growth of *P. yunnanensis*, IAA and ABA increased significantly at early sprouting stage after decapitation, hormones promote tiller growth at different times. Gibberellin and cytokinin can promote the growth and development of lateral branches of Jatropha (Ni et al., 2015), we found that the GA_3_, ZT+GA_3_ and IAA+ZT+GA_3_changed significantly during the development of *P. yunnanensis* sprouts, it indicated that these hormones were involved in the growth and development of lateral branches after decapitation. The change trend of GA_3_ content in this study was similar to that after *Korean pine* was decapitation (Shaolin, 2020). After GA_3_ treatment of tall fescue(cv.’ Barlexas’), CK content decreased and CK degradation gene expression was up-regulated (Zhuang et al., 2019). We found that significant changes in GA_3_ and ZT + GA_3_ after topping promoted the development of lateral branches of *P. yunnanensis*, indicating that there is crosstalk between GA_3_ and ZT, and further research is needed on how the two affect the development of lateral branches in *P. yunnanensis*. Foliar application of GA_3_ stimulated the elongation of main buds and upper primary axillary branches (Mohan Ram and Mehta, 1978), decapitation induces gibberellin synthesis in *Medicago truncatula* to activate axillary bud development (Zhang et al., 2020b), we found that the sprouting mainly occurred in April and May, which may be caused by the significant increase of GA_3_ content in April and May. It was beneficial to relieve the dormancy of axillary buds and promoted the germination of buds. The IAA content of *P. yunnanensis* increased after decapitation, and the IAA content of *Phaseolus vulgaris* also increased after decapitation (Hillman, 1970). The IAA content increased slightly, which may be due to the activation of axillary buds at this time. The continuous growth of buds requires activated buds to maintain auxin synthesis and polar transport (Chabikwa et al., 2019) and promote photosynthesis (Yanhua et al., 2017). We find ABA increased after stubble, and the plant was subjected to mechanical damage as a stress signal to promote the increase of ABA, which was conducive to the plant coping with external interference. After decapitation of broad bean, the ABA in the axillary buds increased first and then decreased, which was similar to our findings (Everat-Bourbouloux and Charnay, 1982). The ABA content of some plants decreased after truncation interference, which may be related to the difference in plant biological characteristics. Decapitation changes the content of endogenous hormones, which in turn causes the relative content and balance of endogenous hormones to change (Chen et al., 1997; Dong et al., 2022). Decapitation significantly increased the balance of GA_3_/ABA, (ZT+GA_3_)/ABA, (IAA+ZT+GA_3_)/ABA during the sprouting process of *P. yunnanensis*. The balance of (IAA+ZT+GA_3_)/ABA reflects the balance between growth-promoting hormones and growth-inhibiting hormones, which can better reflect the growth status of plants. The balance of (IAA+ZT+GA_3_)/ABA after decapitation of *Pinus koraiensis* and *Pinus massoniana* is higher than that non-decapitation (Shaolin, 2020). After *P. yunnanensis* was decapitation, the original hormone balance was broken, the growth-promoting hormones were dominant, and the inhibitory hormones were at a disadvantage, which in turn contributed to the growth of *P. yunnanensis* sprouts.

After decapitation, the content and proportion of *P. yunnanensis* changed, which improved the sprouting ability of the stump. The germination of dormant buds is the result of the redistribution of hormones and nutrients in organs caused by destroying the main shoot apex (Dun et al., 2006; Rinne et al., 2016). The increase of GA can break the dormancy of buds and activate the development of axillary buds (Li et al., 2022). In the study, the significant increase in GA_3_ content was beneficial to break bud dormancy and promoted bud continuous germination of *P. yunnanensis*, and GA_3_ played a more significant role in the emergence of sprouting. GA_3_ played an important role in promoting dormancy breaking and continuous germination of dormant buds after decapitation (Rinne et al., 2016). The activation and growth of axillary buds require gibberellin, which mainly regulates the germination and branching of buds by affecting the differentiation and elongation of cells in buds (Tan et al., 2018). Decapitated hybrid poplar induced GA biosynthesis and *GH17* gene expression, and activated axillary bud germination (Rinne et al., 2016). In perennial trees, plant branching is a complex network regulation, in which gibberellin is a part of plant branching network regulation and plays an important positive role (Fang et al., 2020; Zhang et al., 2020a). This study also found that the number of sprouting branches of *P. yunnanensis* was positively correlated with IAA, ZT, and ABA content, indicating that these three hormones were also involved in the regulation of sprouting branches of *P. yunnanensis*. In the study of *Hippophae rhamnoides* ssp. *sinensis*, it was also confirmed that the number of sprouting tillers after decapitation was significantly positively correlated with IAA content (Kaihong et al., 2018), but auxin is likely to indirectly regulate branching by regulating other hormones (Barbier et al., 2019; Rameau et al., 2014). IAA can regulate the synthesis of GAs and its signal transduction, further synthesis of IAA may increase the content of GAs and indirectly regulate the occurrence of sprouts (Ross et al., 2000). In *Arabidopsis thaliana*, tomato, and Chinese fir, it was found that ABA content was significantly or significantly negatively correlated with the number of sprouts (González-Grandío et al., 2017; Jiling et al., 2021; Wei et al., 2021). In this study, ABA was positively correlated with the number of sprouts, which was different from other research results. However, the correlation coefficient between ABA and the number of sprouts was extremely low, and its effect was negligible. ABA may participate in branching regulation through the relative content of hormones. These results indicated that IAA, ZT, and GA_3_ both positively regulated shoot sprouting and GA_3_ played a leading role in the regulation of shoot sprouting.

The hormone balance is closely related to the sprouting ability of *P. yunnanensis*. The interaction between hormones in plants forms an extremely complex regulatory system, which regulates the growth and development of plants at each stage (Leitão and Enguita, 2016). The dynamic balance between endogenous hormones regulates the metabolism of nucleic acids, proteins and other substances. Thereby controlling the growth and development of plants (Ai et al., 2006; Moore, 1985), and affecting the number of stump sprouts (Assuero and Tognetti, 2010). In this study, the number of sprouts was significantly or significantly positively correlated with GA_3_/ABA, ZT/IAA, (ZT+GA_3_)/ABA, and (IAA+ZT+GA_3_)/ABA. This shows that the hormones IAA, ZT, GA_3,_ and ABA were involved in the regulation process of branching of *P. yunnanensis* through the interaction of hormones, and promotes the occurrence of sprouting by increasing the proportion of hormones. The balance of hormones is more important during the germination of dormant buds, the dynamic balance of GA_3_/ABA is considered to be the key to regulating the dormancy or release of lateral buds (Mornya and Cheng, 2013; Zheng et al., 2015). Increasing GA_3_/ABA can promote the germination of buds, reversal it inhibits its germination (Shaolin, 2020). There was a significant positive correlation between the number of cut-off sprouts and ZT/IAA, and the dynamic balance between IAA and ZT also played an important role in regulating the occurrence of sprouts. The relative change of IAA/CTKs content will promote or inhibit the growth of axillary buds, and then affect the development of plant branches and plant type structure. The balance of CTKs/IAA is positively correlated with the germination of lateral buds after flat stubble. This shows that the interaction between hormones plays an important role in regulating the germination process of buds and is an important factor in the germination of dormant buds.

Hormone content and balance significantly promoted the growth of the sprouting branch of *P. yunnanensis*. The dynamic balance of hormones plays an important role in plant growth and development, ABA/GA balance is essential for seed germination (Jia et al., 2012; Liu et al., 2016). The GA_3_, GA_3_/ABA, (ZT+GA_3_)/ABA, and (IAA+ZT+GA_3_)/ABA were positively correlated with the growth of sprouting branches, indicating that hormone content and dynamic balance played a positive regulatory role in the growth of sprouting branches of *P. yunnanensis*, GA_3_ played a more important role. Gibberellin plays an important role in the formation and size control of various organs during plant vegetative growth (Weiss and Ori, 2007). Higher GA_3_ content and GAs/ABA may be beneficial to the rapid growth of lateral branches after cutting the top (Shaolin, 2020). The GA_3_ content and GA_3_/ABA were significantly or significantly positively correlated with the growth of sprouts (Shi et al., 1992). The balance of (ZR+GA+IAA)/ABA is related to the production and growth of lateral branches, and the increase in the ratio of (IAA+GA+ZR)/ABA will stimulate the growth of lateral branches (QiFeng, 2020). The seedling height growth of the undecapitation was not significantly affected by hormones, while the germination growth of *P. yunnanensis* was significantly regulated by hormones. After decapitation, *P. yunnanensis* promoted compensatory growth by changing hormone content and balance. The growth regulation of control was affected by many factors, the main regulatory factors that need further research.

## 5 Conclusion

*P. yunnanensis* is one of the important forest tree species in Southwest China. Its growth pattern and stand structure have a direct impact on ecosystem function. High-quality panicles that maintain the excellent traits of the mother plant after decapitation can be used for cutting afforestation to improve the stand structure of *P. yunnanensis.* It is urgent to study the changes of hormones after decapitation to promote the occurrence of sprouting. Decapitation had a significant effect on the content and balance of endogenous hormones in *P. yunnanensis*. The contents of GA_3_, ZT+GA_3_, IAA+ZT+GA_3_ and the balances of GA_3_/ABA, (IAA+ZT+GA_3_)/ABA, and (ZT+GA_3_)/ABA were significantly increased during the whole sprouting process by decapitation. The content and proportion of hormones are closely related to the occurrence and growth of sprouting, the dynamic balance of GA_3_ and GA_3_/ABA, (IAA+ZT+GA_3_)/ABA, (ZT+GA_3_)/ABA had more important regulatory effects on the occurrence and growth of *P. yunnanensis* decapitation sprouts. The IAA, ZT, and ABA were also involved in the regulation process of decapitation and promoting sprouts through the interaction of hormones. Hormone content and balance promoted the growth of seedling height in the non-decapition, but the correlation between the two did not reach a significant level. On the whole, after encountering flat stubble interference, *P. yunnanensis* responds to the regulation of endogenous hormone content and ratio balance, and regulates the occurrence and growth of stump sprouting. This study only revealed the physiological mechanism of hormone changes to promote the growth of sprouting after decapitation. Among them, GA_3_ and the interaction between GA_3_ and other hormones played a significant role in promoting the growth of sprouting, which provided a new understanding of the changes in the growth process of sprouting after decapitation. The molecular mechanism of how GA_3_ changes promote the growth of sprouting needs to be further studied. It is a valuable research direction to reveal the changes of GA_3_ from physiological mechanism to molecular mechanism for the growth of sprouting of *P.yunnanensis*.

## Data Availability

The raw data supporting the conclusions of this article will be made available by the authors, without undue reservation.
